# VENTIlatory strategies for patients with severe traumatic brain injury in the LOw- and Middle-Income CountrieS. The VENTILOMICS survey

**DOI:** 10.62675/2965-2774.20250062

**Published:** 2025-07-29

**Authors:** Hemanshu Prabhakar, Charu Mahajan, Indu Kapoor, Gentle S. Shrestha, Edoardo Picetti, Chiara Robba, Marcus J. Schultz, Mani Kalaivani, VENTILOMICS Investigators, Walter Videtta, Gisele Sampaio, Simon P. Gutierrez, Andres M. Rubiano, Manuel Jibaja, Ananya Abate, Yanet Pina Arruebarrena, Tori Sepriwan, Aidos Konkayev, Samuel Ern Hung Tsan, Julio C. Mijangos-Mendez, Chann Myei, Halima M. Salisu-Kabara, Faraz Shafiq, Juan Luis Pinedo, Beda Galicia, Noelia Rivas, Konstantin Popugaev, Llewellyn C. Padayachy, Puvanendiran Shanmugam, Tarig Fadalla, Tanuwong Viarasilpa, Oguzhan ARUN, Peter Kaahwa Agaba, Tuan Van Bui

**Affiliations:** 1 Department of Neuroanaesthesiology and Neurocritical Care All India Institute of Medical Sciences New Delhi India Department of Neuroanaesthesiology and Neurocritical Care, All India Institute of Medical Sciences - New Delhi, India.; 2 Department of Epidemiology and Preventive Medicine Faculty of Medicine, Nursing and Health Sciences Monash University Melbourne Australia Department of Epidemiology and Preventive Medicine, Faculty of Medicine, Nursing and Health Sciences, Monash University - Melbourne, Australia.; 3 Department of Anaesthesia and Intensive Care Parma University Hospital Parma Italy Department of Anaesthesia and Intensive Care, Parma University Hospital, Parma, Italy.; 4 IRCCS Policlinico San Martino Genova Italy Anaesthesia and Intensive Care, IRCCS Policlinico San Martino - Genova, Italy; 5 University of Amsterdam Amsterdam The Netherlands University of Amsterdam - Amsterdam, The Netherlands.; 6 Department of Biostatistics All India Institute of Medical Sciences New Delhi India Department of Biostatistics, All India Institute of Medical Sciences - New Delhi, India.; 1 Department of Intensive Care Posadas Hospital Buenos Aires Argentina Department of Intensive Care, Posadas Hospital - Buenos Aires, Argentina.; 2 Department of Neurology Universidade Federal de São Paulo São Paulo SP Brazil Department of Neurology, Universidade Federal de São Paulo - São Paulo (SP), Brazil; Hospital Israelita Albert Einstein - São Paulo (SP), Brazil.; Hospital Israelita Albert Einstein São Paulo SP Brazil; 3 Hospital de Especialidades Materno Infantile La Paz Bolivia Intensive Care, Hospital de Especialidades Materno Infantile - La Paz, Bolivia.; 4 Neurosciences Institute Universidad El Bosque Bogota Colombia Neurosciences Institute, Universidad El Bosque - Bogota, Colombia; MEDITECH Foundation - Cali, Colombia.; MEDITECH Foundation Cali Colombia; 5 Hospital de Especialidades Eugenio Espejo Escuela de Medicina Universidad San Francisco de Quito Quito Ecuador Unidad de Cuidados Intensivos, Hospital de Especialidades Eugenio Espejo, Escuela de Medicina, Universidad San Francisco de Quito - Quito, Ecuador.; 6 Department of Anesthesiology Addis Ababa University Tikur Anbessa Specialized Hospital Addis Ababa Ethiopia Department of Anesthesiology, Addis Ababa University Tikur Anbessa Specialized Hospital - Addis Ababa, Ethiopia.; 7 Intensive Care Unit Greater Accra Regional Hospital Accra Ghana Intensive Care Unit, Greater Accra Regional Hospital - Accra, Ghana.; 8 Department of Anesthesia and Intensive Therapy Yukum Medical Centre Lampung Indonesia Department of Anesthesia and Intensive Therapy, Yukum Medical Centre - Lampung, Indonesia.; 9 Department of Anesthesia and Intensive Care Astana Medical University Astana Kazakhstan Department of Anesthesia and Intensive Care, Astana Medical University - Astana, Kazakhstan.; 10 Department of Anesthesiology and Intensive Care Faculty of Medicine and Health Sciences University of Malaysia Sarawak Malaysia Department of Anesthesiology and Intensive Care, Faculty of Medicine and Health Sciences, University of Malaysia - Sarawak, Malaysia.; 11 Department of Medical Clinics University Centre for Health Sciences University of Guadalajara Guadalajara Mexico Department of Medical Clinics, University Centre for Health Sciences, University of Guadalajara - Guadalajara, Mexico.; 12 Department of Anaesthesia and Intensive Care Defence Services General Hospital Yangon Myanmar Department of Anaesthesia and Intensive Care, Defence Services General Hospital -Yangon, Myanmar.; 13 Intensive Care Unit Theatres Aminu Kano Teaching Hospital Kano Nigeria Nursing Section Head, Anesthesiology, Intensive Care Unit, Theatres, Aminu Kano Teaching Hospital - Kano, Nigeria.; 14 Department of Anaesthesiology The Aga Khan University Karachi Pakistan Department of Anaesthesiology, The Aga Khan University, Karachi, Pakistan.; 15 Department of Emergency and Critical Care Almanzor Aguinaga Asenjo Hospital Chiclayo Peru Department of Emergency and Critical Care, Almanzor Aguinaga Asenjo Hospital -Chiclayo, Peru.; 16 Nursing Department Specialized in Critical Care Makati Life Medical Centre Manila Philippines Nursing Department Specialized in Critical Care, Makati Life Medical Centre - Manila, Philippines.; 17 Hospital de Clínicas Facultad de Ciencias Médicas Universidad Nacional de Asunción Asunción Paraguay Departamento de Cuidados Intensivos Adultos, Hospital de Clínicas, Facultad de Ciencias Médicas, Universidad Nacional de Asunción - Asunción, Paraguay.; 18 Burnazian State Research Centre Moscow Russia Anaesthesia and Intensive Care, Burnazian State Research Centre - Moscow, Russia.; 19 Department of Neurosurgery Steve Biko Academic Hospital University of Pretoria Pretoria South Africa Brain Tumor and Translational Neuroscience Centre, Department of Neurosurgery, Steve Biko Academic Hospital, University of Pretoria - Pretoria, South Africa.; 20 Intensive Care Unit Peradeniya Teaching Hospital Peradeniya Sri Lanka Intensive Care Unit, Peradeniya Teaching Hospital - Peradeniya, Sri Lanka.; 21 Department of Neurosurgery Soba University Hospital - Khartoum Sudan Department of Neurosurgery, Soba University Hospital - Khartoum, Sudan.; 22 Division of Critical Care Department of Medicine Siriraj Hospital, Mahidol University Bangkok Thailand Division of Critical Care, Department of Medicine, Siriraj Hospital, Mahidol University - Bangkok, Thailand.; 23 Department of Anesthesiology and Reanimation Selcuk University Faculty of Medicine Konya Turkey Department of Anesthesiology and Reanimation, Selcuk University Faculty of Medicine - Konya, Turkey.; 24 Department of Anesthesia and Intensive Care Mulago National Superspecialized Referral Hospital Kampala Uganda Department of Anesthesia and Intensive Care, Mulago National Superspecialized Referral Hospital - Kampala, Uganda.; 25 Neurosurgical Intensive Care Unit Cho Ray Hospital Ho Chi Minh City Vietnam Neurosurgical Intensive Care Unit, Cho Ray Hospital - Ho Chi Minh City, Vietnam.

**Keywords:** Brain injury, traumatic, Critical care, Respiration, artificial, Survey and questionnaires, Developing countries, Economic status, Internationality

## Abstract

**Objective:**

To revisit the VENTIlatory Strategies for Patients with Severe Traumatic Brain Injury (VENTILO) survey, focusing on ventilatory management practices among healthcare professionals in low- and middle-income countries.

**Methodology:**

A cross-sectional on-line survey, VENTIlatory strategies for patients with severe traumatic brain injury in the LOw- and Middle-Income CountrieS (VENTILOMICS), was conducted using the original VENTILO survey questionnaire, developed following a review of literature on respiratory management in traumatic brain injury patients, captured demographics of participants, type of hospital/specialty and available neuromonitoring tools; protocols for mechanical ventilation and weaning, and respiratory management strategies. Descriptive statistics were computed for all study variables. We analyzed data based on the economic status of the low- and middle-income countries.

**Results:**

There were 204 respondents from 28 low- and middle-income countries. Our results indicate that 55 - 70% of respondents recommend tidal volumes of 6 - 8mL/kg for patients with high or medium partial pressure of arterial oxygen/inspired fraction of oxygen, while tidal volumes of 4 - 6mL/kg is preferred for those with low partial pressure of arterial oxygen/inspired fraction of oxygen ratios. For patients with intracranial hypertension, lower positive end-expiratory pressure levels were utilized.

**Conclusion:**

The findings suggest a consistent approach to lung-protective ventilation across low-and middle-income countries, with notable variations influenced by local resources and economic status. This study highlights the necessity for tailored research and guidelines to address the specific challenges faced in traumatic brain injury management within low-and middle-income countries.

## INTRODUCTION

Traumatic brain injury (TBI) remains a leading global neurological problem causing significant socioeconomic and healthcare burden.^1^About 28 million people sustain a TBI each year, with 50 million currently living with it, causing over 7 million years of disability.^2^ This burden is even greater in low- and middle-income countries (LMICs), where limited resources and prevention measures, like helmet use, contribute to higher rates of TBI.^3,4^ Patients sustaining severe TBI often require artificial airways to protect airways and invasive mechanical ventilation (MV) to maintain oxygenation and carbon dioxide clearance.^5^ Subsequently, this may lead to respiratory complications, as head-injured patients with endotracheal tubes are particularly susceptible to the development of acute respiratory distress syndrome (ARDS).^6^

Ventilatory management of patients sustaining TBI is challenging because many currently advocated ventilation strategies like lung-protective ventilation, which protects alveoli and may be adjusted to target a desired carbon dioxide clearance, and permissive hypercapnia can adversely affect the brain by causing cerebral vasodilation and increasing cerebral blood volume. Furthermore, there is a lack of sufficient literature on optimal ventilatory strategies and targets for ventilation in patients with TBI.^7-10^

Aiming to explore respiratory management practices in TBI patients, the VENTIlatory Strategies for Patients with Severe Traumatic Brain Injury (VENTILO) investigators performed survey a few years ago.^11^ This survey, endorsed by the European Society of Intensive Care Medicine (ESICM), showed important differences in respiratory management practices in TBI patients. However, most of the respondents to this survey were from Europe. Representation from the LMICs was limited; Sudan, Brazil, India, and Ukraine were the main ones represented. Therefore, we decided to revisit this survey to assess ventilatory management practice in TBI patients in the LMICs.

## METHODS

### Study design and ethics approval

This was a cross-sectional on-line survey. Ethical approval for this survey was obtained from Institute Ethics Committee (IEC), before the survey started.

### Survey

The original VENTILO survey,^11^ developed following a review of literature on respiratory management in TBI patients, captured participants’ demographics, type of hospital/specialty and available neuromonitoring tools; protocols for MV and weaning; and respiratory management strategies. In the present survey, named VENTIlatory strategies for patients with severe traumatic brain injury in the LOw- and Middle-Income CountrieS (VENTILOMICS), the questionnaire was revised and modified by two investigators, keeping in mind the context and resource-limited settings in the LMICs. Eight new questions were added to the original questionnaire as they felt relevant in the LMICs. Once the questionnaire was finalized, we performed a pilot survey testing among five of our experts in the writing group. This was performed to identify and fix issues prior to the survey being launched to a larger group.

Respondents were asked about their area of practice (rural/urban). They were also enquired about the use of the prone position during MV in patients with TBI and what sedatives, analgesics, and muscle relaxants they used. They were also questioned about their preference for early *versus* late tracheostomy.

### Participants

The participants of this international survey included all healthcare workers involved in the care of TBI patients across various LMICs. We allowed more than one response from different respondents in the same hospital, considering that different experts would have different management strategies, even in the same department. We did not exclude any respondents who completed the questionnaire.

### Survey distribution

This survey was initially distributed to representatives (VENTILOMICS Investigators) from the different LMICs, who were considered experts (physicians and healthcare workers) in their field and involved in the daily care of TBI patients. These representatives have good clinical experience and academic interests and were also involved in research. Participants were recruited through emails sent by representatives from the various LMICs. The survey link was also shared on social media such as WhatsApp and Twitter. The survey was carried out over 3 months, from March 2024 to May 2024. Study data were collected and managed using Google Forms. The collected information was automatically entered into an Excel spreadsheet.

### Questionnaire

An electronic survey composed of 38 items and 3 different clinical scenarios (partial pressure of arterial oxygen [PaO_2_]/inspired fraction of oxygen [FiO_2_] > 300, 150 - 300, and < 150) was used, revising and modifying the VENTILO survey.^11^We considered PaO_2_/FiO_2_ of > 300, between 150 - 300 and < 150 as high, medium and low PaO_2_/FiO_2_, respectively. Since these cutoffs differ from those used for severity classification, we did not categorize them as mild, moderate, or severe ARDS^12^ (Survey questionnaire - Supplementary Material).

### Data storage

Data were collected and stored as spreadsheets in a secure Google database. Only one author had access to data to respect respondents’ confidentiality and anonymity.

### Statistical analysis

#### Data from the questionnaire was stored as an Excel file (Microsoft Corp, Redmond, WA).

Stata 18.0 (StataCorp LP, Texas, USA) was used for statistical analysis. Descriptive statistics were computed for all study variables. We analyzed data based on their economic status [low-income country (LIC), lower middle-income country (LoMIC), and upper middle-income country (UMIC)]. We also analyzed data based on the characteristics of intensive care unit (ICU), such as general or neurocritical care ICU (NICU). The results are presented as number (%). Differences in the groups were assessed using the Chi-squared test. Given the exploratory nature of this study, a priori sample size calculations and power analyses were not conducted. All p values were two-tailed, with values below 0.05 considered statistically significant.

## RESULTS

### Respondents

**Figure 1:**
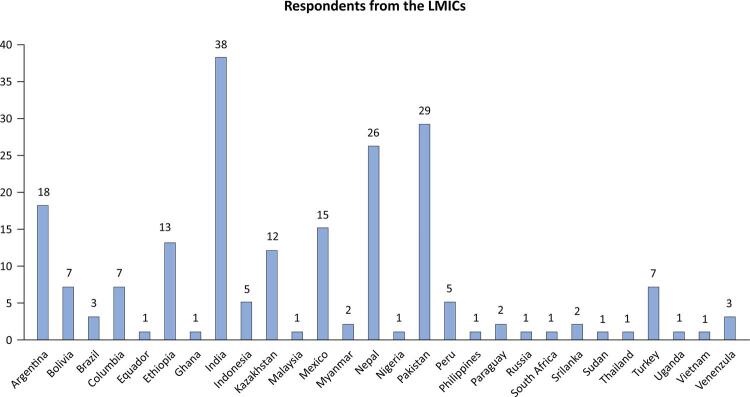
Number of respondents from different low-and middle-income countries. LMICS - low-income and middle-income countries.

The survey included 204 respondents from 28 LMICs (Figure 1S - Supplementary Material), with most respondents from India, Pakistan, and Nepal (Figure 1). The majority were intensivists, neuro-intensivists, or anesthesiologists working in general or specialized neuro-ICUs (Table 1). Most respondents (178/204 (87.3%) had not participated in the previous VENTILO survey. We did not exclude any response from the final analysis.

**Table 1:** Baseline characteristics of the respondents

	Respondents
Gender	
Male	128 (62.7)
Female	76 (37.3)
Age (years)	
< 35	63 (30.9)
36 - 45	75 (36.8)
46 - 60	57 (27.9)
> 60	9 (4.4)
Specialty	
Anesthesia	98 (48)
Intensive care medicine	125 (61.3)
Neurointensive care	54 (26.5)
Neurology	4 (2)
Neurosurgery	10 (4.9)
General physician	20 (9.8)
Pulmonary/respiratory	12 (5.9)
Area of practice	
Urban	198 (97.1)
Rural	6 (2.9)
Professional category	
Consultant doctor	148 (72.5)
Resident doctor	40 (19.6)
Nurse	6 (2.9)
Respiratory therapist	10 (4.9)
Post specialization experience (years)	
1 - 5	82 (40.2)
6 - 10	30 (14.7)
11 - 15	33 (16.2)
> 15	59 (28.9)
Type of ICU	
General ICU	158 (77.5)
Neuro ICU	46 (22.5)
ICU beds	
< 5	39 (19.1)
6 - 10	87 (42.6)
11 - 15	37 (18.1)
> 15	41 (20.1)
Affiliation	
Government teaching	119 (58.3)
Government non-teaching	9 (4.4)
Private teaching	47 (23)
Private non-teaching	29 (14.2)
Available bedside monitoring	
ICP	107 (52.5)
NIRS	42 (20.6)
PbtO_2_	22 (10.8)
TCD	79 (38.7)
Cerebral microdialysis	6 (2.9)
Intermittent EEG	118 (57.8)
Continuous EEG	54 (26.5)
Automated pupillometer	28 (13.7)
SjVO_2_	46 (22.5)
POCUS	140 (68.6)

ICU - intensive care unit; ICP - intracranial pressure; NIRS - near-infrared spectroscopy; PbtO_2_ - brain tissue oxygenation; TCD - transcranial Doppler; EEG - electroencephalograph; SjVO_2_ - jugular venous oximetry; POCUS - point of care ultrasound. Results are presented as n (%).

### Recommendations for tidal volume

For patients with high or medium PaO_2_/FiO_2_, the majority of respondents (approximately 55 - 70% respondents) suggested that the setting of tidal volume (V_T_) was between 6 - 8mL/kg predicted body weight (PBW). The most recommended setting for V_T_ in patients with a low PaO_2_/FiO_2_ was 4 - 6mL/kg PWB (Table 2).

**Table 2:** Ventilator settings and respiratory targets utilized in the three clinical settings

PaO_2_/FiO_2_	TV (mL/kg PBW)	Highest PEEP No IH (cmH_2_O)	Highest PEEP IH (cmH_2_O)	PaCO_2_ No IH (mmHg)	PaCO_2_ IH (mmHg)	PaO_2_ (mmHg)	SpO_2_ (%)
> 300	4 - 6 [46 (22.5)]	5 [36 (17.7)]	5 [88 (43.1)]	30 - 35 [31 (15.2)]	30 - 35 [120 (58.8)]	55 - 80 [37 (18.1)]	88 - 91 [9 (4.4)]
	6 - 8 [46 (22.5)]	8 [49 (24)]	8 [58 (28.4)]	36 - 40 [132 (64.7)]	36 - 40 [73 (35.8)]	81 - 100 [109 (53.4)]	92 - 94 [99 (48.5)]
	8 - 10 [10 (4.9)]	10 [61 (29.9)]	10 [49 (24)]	41 - 45 [30 (14.7)]	41 - 45 [7 (3.4)]	101 - 120 [34 (16.7)]	> 95 [96 (47.1)]
	> 10 [1 (0.5)]	15 [58 (28.4)]	15 [9 (4.4)]	46 - 55 [11 (5.4)]	46 - 55 [4 (1.9)]	> 120 [8 (3.9)]	
					† Adj [16 (7.8)		
150 - 300	4 - 6 [83 (40.7)]	5 [19 (9.3)]	5 [53 (25.9)]	30 - 35 [27(13.21)]	30 - 35 [102 (50)]	55 - 80 [42 (20.61)]	88 - 91[20 (9.8)]
	6 - 8 [114 (55.9)]	8 [36 (17.7)]	8 [68 (33.3)]	36 - 40 [119 (58.3)]	36 - 40 [90 (44.1)]	81 - 100 [112 (54.9)]	92 - 94 [117 (57.4)]
	8 - 10 [6 (2.9)]	10 [74 (36.3)]	10 [68 (33.3)]	41 - 45 [44 (21.6)]	41 - 45 [8 (3.9)]	101 - 120 [34 (16.7)]	> 95 [64 (32.8)]
	> 10 [1 (0.5)]	15 [75 (36.8)]	15 [15 (7.4)]	46 - 55 [14 (6.7)]	46 - 55 [1(0.5)]	> 120 [4 (1.9)]	
					*Adj [3 (1.5)]	† Adj [12 (5.9)]	
< 150	4 - 6 [134 (65.7)]	5 [15 (7.4)]	5 [39 (19.1)]	30 - 35 [21 (10.3)]	30 - 35 [84 (41.2)]	55 - 80 [61 (29.9)]	88 - 91 [59 (28.9)]
	6 - 8 [61 (29.9)]	8 [26 (12.8)]	8 [61 (29.9)]	36 - 40 [104 (50.9)]	36 - 40 [83 (40.7)]	81 - 100 [104 (50.9)]	92 - 94 [97 (47.6)]
	8 - 10 [7 (3.4)]	10 [64 (31.4)]	10 [71 (34.8)]	41 - 45 [45 (22.1)]	41 - 45 [27 (13.2)]	101 - 120 [22 (10.8)]	> 95 [48 (23.5)]
	> 10 [2 (0.9)]	15 [99 (48.5)]	15 [33 (16.2)]	46 - 55 [15 (7.4)]	46 - 55 [4 (1.9)]	> 120 [5 (2.5)]	
				*Adj [19 (9.3)]	*Adj [6 (2.9)]	*Adj [12 (5.9)]	

PaO_2_ - partial pressure of arterial oxygen; FiO_2_ - fraction of inspired oxygen; TV - tidal volume; PBW - predicted body weight; PEEP - positive end-expiratory pressure; IH - intracranial hypertension; PaCO_2_ - partial pressure of arterial carbon dioxide; SpO_2_ - oxygen saturation. *Adj - adjusted (any PaCO_2_ if pH is within normal range); †Adj - adjusted (tailored to neuromonitoring data).

### Recommendations for patients without intracranial hypertension

In patients with medium or low PaO_2_/FiO_2_, the frequently utilized upper positive end-expiratory pressure (PEEP) level was 15cmH_2_O [n = 75 (37%) and 99 (48.5%)], respectively; among those with high PaO_2_/FiO_2_, the upper PEEP level of 10cmH_2_O was the most utilized [n = 61 (30%)]. The respiratory targets most frequently employed, irrespective of the PaO_2_/FiO_2_, were partial pressure of arterial carbon dioxide (PaCO_2_) 36 - 40mmHg, PaO_2_ 81 - 100mmHg, and oxygen saturation (SpO_2_) 92 - 94%.

### Recommendations for patients with intracranial hypertension

While V_T_ recommendations were similar to those in patients without intracranial hypertension (Table 2), our survey (43% respondents) suggested lower upper PEEP levels of up to 5cmH_2_O for patients with high PaO_2_/FiO_2_; 8 to 10cmH_2_O for patients with medium PaO_2_/FiO_2_, and 10cmH_2_O for patients with low PaO_2_/FiO_2_. The PaCO_2_ targets (30 - 35mmHg) were identical for all PaO_2_/FiO_2_, and PaO_2_ and SpO_2_ targets remained unchanged, that is, 81 to 100mmHg for PaO_2_ and 92 - 94% for SpO_2_, irrespective of the PaO_2_/FiO_2_.

### Comparison of the intensive care units

Respondents from neuro-ICUs had better access to intracranial pressure (ICP) monitors (87%), near-infrared spectroscopy (NIRS) (46%), transcranial Doppler (TCD) (76%), continuous electroencephalogram (EEG) (48%) and automated pupillometer (39%) compared to those from general ICUs (Table 3). The use of automated ventilation modes (a type of MV in which the ventilator adjusts settings dynamically in response to the patient’s physiological parameters, aiming to optimize ventilation and reduce the need for manual adjustments by the clinician), protocols for MV and weaning, target driving and plateau pressures, thresholds for using prone position in respiratory failure, medications used during prone MV and tracheostomy plans were comparable in both, neuro-ICUs and general ICUs (Table 3).

**Table 3:** Comparison between data from general and neurocritical care intensive care units

	General ICU (n = 158)	Neuro-ICU (n = 40)	p value
Specialty			
Anesthesia	73 (46.2)	25 (54.4)	0.33
Intensive care medicine	104 (65.8)	(45.7)	0.01
Neurointensive care	29 (18.4)	25 (54.4)	< 0.01
Neurology	3 (1.9)	1 (2.2)	0.91
Neurosurgery	6 (3.8)	4 (8.7)	0.18
General physician	19 (12.0)	1 (2.2)	0.05
Pulmonary/respiratory	10 (6.3)	2 (4.4)	0.62
Available bedside monitoring			
ICP	67 (42.4)	40 (86.9)	< 0.01
NIRS	21 (13.3)	21 (45.7)	< 0.01
PbtO_2_	14 (8.9)	8 (17.4)	0.10
TCD	44 (27.9)	35 (76.1)	< 0.01
Cerebral microdialysis	5 (3.2)	1 (2.2)	0.73
Intermittent EEG	89 (56.3)	29 (63.0)	0.42
Continuous EEG	32 (20.3)	22 (47.8)	< 0.01
Automated pupillometer	10 (6.3)	18 (39.1)	< 0.01
SjVO_2_	34 (21.5)	12 (26.1)	0.51
POCUS	103 (65.2)	37 (80.4)	0.05
Protocol for MV	101 (63.9)	35 (76.1)	0.12
Parameters in protocol			
Tidal volume	99 (62.7)	33 (71.7)	0.26
PEEP	90 (56.9)	32 (69.6)	0.13
FiO_2_	92 (58.2)	35 (76.1)	0.03
Respiratory rate	87 (55.1)	31 (67.4)	0.14
Driving pressure	71 (44.9)	23 (50)	0.54
Plateau pressure	80 (50.6)	31 (67.4)	0.05
Transpulmonary pressure	17 (10.8)	2 (4.4)	0.19
Mechanical power	24 (15.2)	7 (15.2)	0.99
Use of automated ventilation modes	87 (55.1)	23 (50)	0.54
Protocol for weaning present	127 (80.4)	39 (84.8)	0.50
Parameters for weaning			
pH	85 (53.8)	33 (71.7)	0.03
Glasgow coma scale	128 (81)	40 (86.9)	0.35
PaO_2_/FiO_2_	121 (76.6)	40 (86.9)	0.13
PaO_2_	78 (49.4)	30 (65.2)	0.06
PaCO_2_	88 (55.7)	32 (69.6)	0.09
Strength of cough	117 (74.1)	39 (84.8)	0.13
Swallowing	97 (61.4)	34 (73.9)	0.12
Tracheal secretions quantity	97 (61.4)	34 (73.9)	0.12
Target driving pressure < 15cmH_2_O	138 (87.3)	36 (78.3)	0.13
Target plateau pressure < 30cmH_2_O	46 (92.4)	43 (93.5)	0.81
Thresholds for a prone position in respiratory failure			
PaO_2_/FiO_2_ < 100mmHg	102 (64.6)	23 (50)	
PaO_2_/FiO_2_ 100 - < 200mmHg	50 (31.7)	21 (45.7)	
PaO_2_/FiO_2_ > 200 - < 300mmHg	6 (3.8)	2 (4.4)	0.19
Position of bed during prone ventilation			
Flat	69 (43.7)	18 (39.1)	
Reverse trendelenburg	89 (56.3)	28 (60.9)	0.58
Medications during prone MV			
Midazolam	90 (56.9)	31 (67.4)	0.21
Propofol	121 (76.6)	33 (71.7)	0.50
Dexmedetomidine	68 (43.0)	21 (45.7)	0.75
Fentanyl	128 (81)	38 (82.6)	0.81
Remifentanil	26 (16.5)	9 (19.6)	0.62
Rocuronium	42 (26.8)	18 (39.1)	0.11
Atracurium	74 (46.8)	21 (45.7)	0.89
Cis-Atracurium	56 (35.4)	15 (32.6)	0.72
Tracheostomy plan			
Early (< 7days)	99 (62.7)	34 (73.9)	
Late (> 7 days)	59 (37.3)	12 (26.1)	0.16

ICU - intensive care unit; ICP - intracranial pressure; NIRS - near infrared spectroscopy; PbtO_2_ - brain tissue oxygenation; TCD - Transcranial Doppler; EEG - electroencephalograph; SjVO_2_ - jugular venous oximetry; POCUS - point of care ultrasound; MV - mechanical ventilation; PEEP - positive end-expiratory pressure; FiO_2_ - fraction of inspired oxygen; pH - potential of hydrogen; PaO_2_ - partial pressure of arterial oxygen; PaCO_2_ - partial pressure of arterial carbon dioxide. Results are presented as n (%).

### Geoeconomic differences

Most respondents from the LICs were anesthesiologists (93%), while those from the UMIC were intensivists (73%) (Table 4). Neurologic and neurosurgical patients were mostly managed in general ICUs. There was significant variation across the geoeconomic regions in the presence of ventilation protocols and their specific recommendations (65% and 71% in the LoMIC and UMIC, respectively, *versus* only 27% in the LICs). The presence of weaning protocols was not different, but recommendations for weaning were different. Compared to respondents from UMICs and LoMICs, respondents from LICs had limited or negligible access to ICP monitors, NIRS, TCD, intermittent electroencephalogram (iEEG), jugular venous oximetry (SjVO_2_), and point of care ultrasound (POCUS) (Table 4).

**Table 4:** Comparison of data between various low - income and middle-income countries based on their economic status

	LIC (n = 15)	LoMIC (n= 107)	UMIC (n = 82)	p value
Gender				
Male	11 (73.3)	64 (59.8)	53 (64.6)	
Female	4 (26.7)	43 (40.2)	9 (35.4)	0.54
Age				
< 35	11 (73.3)	40 (37.4)	12 (14.6)	
36 - 45	4 (26.7)	37 (34.6)	34 (41.5)	
46 - 60	0 (0)	25 (23.4)	32 (39)	
> 60	0 (0)	5 (4.7)	4 (4.9)	< 0.01
Specialty				
Anesthesia	14 (93.3)	62 (57.9)	22 (26.8)	< 0.01
Intensive care medicine	4 (26.7)	61 (57)	60 (73.2)	< 0.01
Neurointensive care	1 (6.7)	29 (27.1)	24 (29.3)	0.19
Neurology	0 (0)	2 (1.9)	2 (2.4)	0.82
Neurosurgery	0 (0)	3 (2.8)	7 (8.5)	0.13
General physician	1 (6.7)	17 (15.9)	2 (2.4)	0.01
Pulmonary/respiratory	0 (0)	8 (7.5)	4 (4.9)	0.45
Professional category				
Consultant doctor	14 (93.3)	64 (59.8)	70 (85.4)	
Resident doctor	1 (6.7)	28 (26.2)	11 (13.4)	
Nurse	0 (0)	6 (5.6)	6 (6)	
Respiratory therapist	0 (0)	9 (8.4)	1 (1.2)	< 0.01
Post specialization experience (years)				
1 - 5	14 (93.3)	51 (47.7)	17 (20.7)	
6 - 10	0 (0)	15 (14)	15 (18.3)	
11 - 15	1 (6.7)	17 (15.9)	5 (18.3)	
> 15	0 (0)	24 (22.4)	35 (42.7)	< 0.01
Type of ICU				
General ICU	15 (100)	77 (71.9)	66 (80.5)	
Neuro ICU	0 (0)	30 (28)	16 (19.5)	0.04
ICU beds				
< 5	8 (53.3)	16 (14.9)	15 (18.3)	
6 - 10	7 (46.7)	43 (40.2)	37 (45.1)	
11 - 15	0 (0)	19 (17.8)	18 (21.9)	
> 15	0 (0)	29 (27.1)	12 (14.6)	< 0.01
Affiliation				
Government teaching	12 (80)	51 (47.7)	56 (68.3)	
Government non-teaching	1 (6.7)	1 (0.9)	7 (8.5)	
Private teaching	0 (0)	35 (32.7)	12 (14.6)	
Private non-teaching	2 (13.3)	20 (18.7)	7 (8.5)	< 0.01
Available bedside monitoring				
ICP	0 (0)	60 (56.1)	47 (57.3)	< 0.01
NIRS	0 (0)	20 (18.7)	22 (26.8)	0.05
PbtO_2_	2 (13.3)	8 (7.5)	12 (14.6)	0.23
TCD	1 (6.7)	46 (42.9)	32 (39)	0.03
Cerebral microdialysis	0 (0)	2 (1.9)	4 (4.9)	0.38
Intermittent EEG	3 (20)	61 (57)	54 (65.9)	< 0.01
Continuous EEG	1 (6.7)	30 (28)	23 (28.1)	0.19
Automated pupillometer	1 (6.7)	18 (16.8)	9 (10.9)	0.36
SjVO_2_	0 (0)	12 (11.2)	34 (41.5)	< 0.01
POCUS	13 (86.7)	78 (72.9)	49 (59.8)	0.05
Protocol for MV present	4 (26.7)	79 (73.8)	53 (64.6)	< 0.01
Parameters in protocol				
Tidal volume	4 (26.7)	76 (71)	52 (63.4)	< 0.01
PEEP	4 (26.7)	72 (67.3)	46 (56.1)	0.01
FiO_2_	4 (26.7)	73 (68.2)	0 (60.9)	0.01
Respiratory rate	4 (26.7)	69 (64.5)	45 (54.9)	0.02
Driving pressure	1 (6.7)	51 (47.7)	42 (51.2)	0.01
Plateau pressure	4 (26.7)	67 (62.6)	40 (48.8)	0.01
Transpulmonary pressure	0 (0)	12 (11.2)	7 (8.5)	0.36
Mechanical power	0 (0)	15 (14)	16 (19.5)	0.14
Use of automated ventilation modes	9 (60)	52 (48.6)	49 (59.8)	0.28
Protocol for weaning present	11 (73.3)	89 (83.2)	66 (80.5)	0.63
Parameters for weaning				
pH	1 (6.7)	70 (65.4)	47 (57.3)	< 0.01
Glasgow coma scale	13 (86.7)	94 (87.9)	61 (74.4)	0.05
PaO_2_/FiO_2_	3 (20)	86 (80.4)	72 (87.8)	< 0.01
PaO_2_	4 (26.7)	65 (60.8)	39 (47.6)	0.02
PaCO_2_	1 (6.7)	73 (68.2)	46 (56.1)	< 0.01
Strength of cough	15 (100)	77 (71.9)	64 (78.1)	0.05
Swallowing	10 (66.7)	65 (60.8)	56 (68.3)	0.55
Tracheal secretions quantity	13 (86.7)	58 (54.2)	60 (73.2)	0.01
Target driving pressure < 15cmH_2_O	12 (80)	89 (83.2)	73 (89)	0.44
Target plateau pressure < 30cmH_2_O	14 (93.3)	102 (95.3)	73 (89)	0.23
Thresholds for a prone position in respiratory failure				
PaO_2_/FiO_2_ < 100mmHg	12 (80)	68 (63.6)	45 (54.9)	
PaO_2_/FiO_2_ 100 - < 200mmHg	2 (13.3)	33 (30.8)	36 (43.9)	
PaO_2_/FiO_2_ > 200 - < 300mmHg	1 (6.7)	6 (5.6)	1 (1.2)	0.08
Position of bed during prone ventilation				
Flat	4 (26.7)	46 (42.9)	37 (45.1)	
Reverse trendelenburg	11 (73.3)	61 (57)	45 (54.9)	0.41
Medications during prone MV				
Midazolam	7 (46.7)	70 (65.4)	44 (53.7)	0.16
Propofol	15 (100)	73 (68.2)	66 (80.5)	0.01
Dexmedetomidine	8 (53.3)	48 (44.9)	3 (40.2)	0.6
Fentanyl	14 (93.3)	85 (79.4)	67 (81.7)	0.43
Remifentanil	6 (40)	10 (9.4)	19 (23.2)	0.01
Rocuronium	2 (14.3)	20 (18.7)	38 (46.3)	< 0.01
Atracurium	7 (46.7)	66 (61.7)	22 (26.8)	< 0.01
Cis-Atracurium	7 (46.7)	42 (39.3)	22 (26.8)	0.13
Tracheostomy plan				
Early (< 7days)	7 (46.7)	80 (74.8)	46 (56.1)	
Late (> 7 days)	8 (53.3)	27 (25.2)	36 (43.9)	0.01

LIC - low-income countries; LoMIC - lower-middle-income countries; UMIC - upper middle-income countries; ICU - intensive care unit; ICP - intracranial pressure; NIRS - near-infrared spectroscopy; PbtO_2_ - brain tissue oxygenation; TCD - transcranial Doppler; EEG - electroencephalograph; SjVO_2_ - jugular venous oximetry; POCUS - point of care ultrasound; MV - mechanical ventilation; PEEP - positive end-expiratory pressure; FiO_2_ - fraction of inspired oxygen; pH - potential of hydrogen; PaO_2_ - partial pressure of arterial oxygen; PaCO_2_ - partial pressure of arterial carbon dioxide. Results are presented as n (%).

### Rescue strategies

**Figure 2:**
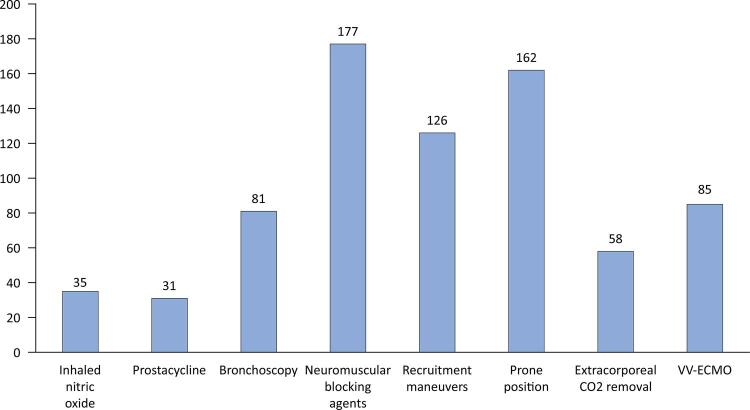
Rescue strategies used in respiratory failure. CO_2_ - carbon dioxide; VV- ECMO – Venovenous extracorporeal membrane oxygenator

In case of refractory respiratory failure, neuromuscular blocking agents (NMBAs) are the commonest rescue strategy, followed by prone positioning (80%) and recruitment maneuvers (62%) (Figure 2).

## DISCUSSION

This international survey, conducted across various LMICs, provides important insight regarding the respiratory management of patients with TBI who have been admitted to the ICU.

### Ventilator settings and respiratory targets in traumatic brain injury

The second hit theory of brain-lung crosstalk highlights the deleterious mechanical effect of ventilation on the injured brain.^13^ The V_T_ close to 6 - 8mL/kg of PBW is strongly recommended in several trials and systematic reviews.^12,14^ The beneficial effects of lung protective ventilation (LPV) are observed in patients with and without ARDS, making it a general practice.^15^ The protective MV strategy for patients with concomitant acute brain injury and ARDS includes using low V_T_ (4 - 8mL/kg of PBW) and low minute volume, avoiding permissive hypercapnia. In our study, most respondents also chose V_T_ of 6 - 8mL/kg for patients with medium PaO_2_/FiO_2_. In our survey, 65.7% of respondents chose a lower V_T_ of 4 - 6mL/kg in patients with a low PaO_2_/FiO_2_. This is slightly higher than the findings of the VENTILO survey, in which only 53% of respondents use V_T_ 4 - 6mL/kg in patients with a PaO_2_/FiO_2_ < 150.^11^ In a recent trial, the effects of low V_T_ with low PEEP (< 5cmH_2_O) and low V_T_ with high PEEP (> 12cmH_2_O) on ICP and oxygenation were studied. The authors found that an increase in PEEP did not influence ICP.^16^ However, 15% of the attempted interventions in six patients were stopped because ICP increased to more than 22mmHg for 5 minutes. It is suggested that though LPV seems safe, its effect depends on baseline ICP, brain compensatory reserve, and mechanical power.^16^

The ESICM Guidelines on MV in brain-injured patients strongly recommend maintaining a PaO_2_ of 80 - 120mmHg in all acute brain-injured patients, irrespective of the ICP.^(8)^ Most respondents in our survey target a PaO_2_ of 81 - 100mmHg while managing patients with TBI, similar to the findings of the VENTILO Survey.^11^ In patients with brain hypoxia (PbtO_2_ levels < 20mmHg), it is recommended to increase the FiO_2_ to 0.6 in tier one; and to target PaO_2_ of 150mmHg and above in tier 2 - 3, respectively.^17^ However, in the LMICs, the availability of probes is a major limiting step (utilized only by 10.8% of our respondents). The availability of sophisticated monitors is an important reason patients are transferred to referral centers or those with sophisticated monitoring systems. The non-availability of monitoring could be the reason for using higher oxygen levels, which may induce hyperoxia-induced injury. Another important consideration is the limited availability of respiratory therapists in the LMICs.

Most respondents in our survey target a PaCO_2_ of 36 - 40mmHg in daily clinical practice. However, in patients with intracranial hypertension, a slightly lower range (30 - 35mmHg) was preferred by the majority of responders, which is in contrast with the data of the VENTILO survey,^11^ in which a PaCO_2_ of 36 - 40mmHg was mainly chosen even in the presence of intracranial hypertension.

In patients with intracranial hypertension with ARDS, we found that the majority of respondents agree that a PEEP of 10cmH_2_O can be applied to maintain oxygenation. Multimodal neuromonitoring (ICP and brain tissue oxygenation) can help individualize PEEP and maintain it below ICP based on cerebral and lung compliance.^18,19^

### Protocols and modes of mechanical ventilation

In the VENTILO survey,^11^ a less frequent use of standardized protocol for MV and weaning, when comparing responses from the European *versus* non-European respondents, was reported. We found that protocols less frequently used in the LIC. However, nearly 70 - 80% of respondents in our survey utilize a standardized protocol for weaning. The utilization of automated ventilation modes was comparable across the various LMICs, ranging from 45 - 70%, almost similar to the VENTILO survey.

### Rescue strategies utilized in refractory respiratory failure

Refractory respiratory failure (PaO_2_/FiO_2_ < 150mmHg) remains a challenge in TBI patients where protocols of general ICU cannot always applied. The first strategy used by our respondents is the use of NMBAs, similar to the VENTILO survey.^11^ It has been suggested that if a short trial of NMBAs in ARDS patients with ventilator dyssynchrony shows improvement in PaO_2_, it may be continued, preferably for less than 48 hours.^20^ In this context, the Seattle International Severe Traumatic Brain Injury Consensus Conference (SIBICC) protocol suggests a short trial of NMBAs as tier two intervention in case of raised ICP.^17^

Prone position causes alveolar recruitment, improves oxygenation, and reduces mortality in severe ARDS.^21^ We found that 79.4% of respondents would use the prone position in our survey compared to 63% in the VENTILO survey.^11^

A recent systematic review studied the feasibility of prone positioning in brain-injured patients with severe ARDS.^22^ The authors found that 75% of the selected studies excluded patients with brain injuries and that the number of patients recorded from randomized controlled trials was too small to carry out a meta-analysis. The results, however, indicate an increase in ICP after 1 hour of prone position. The small number of patients included prevented the authors from making any recommendations.

Our survey shows that about 62% of respondents would use recruitment maneuvers, comparable to the 69% reported in the VENTILO survey.^11^ The use of recruitment maneuvers can increase intrathoracic pressure and hamper cerebral venous return, thereby reducing cerebral perfusion pressure and causing deleterious effects in brain-injured patients (especially in the case of deranged cerebral autoregulation). If hemodynamic stability is maintained, it can be safely used if associated with improved oxygenation.^18,23^ However, it must be mentioned that the updated guidelines on the management of adult patients with ARDS recommend against the use of lung recruitment maneuvers.^24^

The extracorporeal membrane oxygenation (ECMO) can potentially be a life-saving modality in hypoxemic patients with severe TBI. However, anticoagulation associated with the procedure can promote the progression of hemorrhagic lesions.^24^ The huge human and financial burden can be a limiting step in its widespread use in resource-limited countries. Notably, 41% of our respondents from LMICs utilize venovenous ECMO, almost comparable to 47% in the VENTILO survey.^11^ The high use of ECMO in our survey suggests a respondent bias as sophisticated equipment may not be available uniformly in the LMICs. These findings are, therefore, not generalizable.

### Strengths

An important strength of our study is its international representation. There are experts and respondents from 28 LMICs in this study. No previous study has provided data from the LMICs based on their economic status. Our study gives valuable insight into the variations of practice across the LMICs. The variation between practitioners working in general and neuro-ICUs helps identify gaps due to limited resources. The difference in the economic status of the LMICs, resulting in variations in practice, is also identified in this study; this has never been addressed in any previous trial. Information collected in this study can help in planning future research in the LMICs.

### Limitations

This study presents some limitations. Firstly, surveys have inherent drawbacks, such as inflexibility and validity. It is challenging to calculate survey response rates, mainly if they are circulated through social media groups and emails. Our respondents worked in either NICUs or general ICUs. This article discusses only the respiratory management of patients with TBI and not the patients’ other systemic or specific clinical conditions. We did not use the defined cut-off values for PaO_2_/FiO_2_ in ARDS as we wanted to remain close to the original survey because we used the same clinical scenarios as those in the VENTILO study.^11^ Another limitation can be a lack of proportionate representation from various nations/regions and within the nation (various set-ups within a nation). This would lead to a likely failure to elicit the exact scenarios in all the LMICs. A limitation of this study is that only a single participant represented some countries. There was also a low representation form LICs (15 of 204 respondents). This limits the ability to draw reliable conclusions about national clinical practices in those countries. Our respondents were physicians and nurses, so respondent bias cannot be ignored. Therefore, some of our findings may not be generalizable.

## CONCLUSION

The international survey showed variation in ventilatory management of head-injured patients across different geographical regions. This study highlights the necessity for tailored research and guidelines to address the specific challenges faced in traumatic brain injury management within low-and middle-income countries, thereby advocating improved access to neuromonitoring and standardized protocols.
